# Performance Comparison of Two Sensors Based on Surface Plasmon Resonance in a Plastic Optical Fiber

**DOI:** 10.3390/s130100721

**Published:** 2013-01-07

**Authors:** Nunzio Cennamo, Davide Massarotti, Ramona Galatus, Laura Conte, Luigi Zeni

**Affiliations:** 1 Department of Information Engineering, Second University of Naples, Via Roma 29, 81031 Aversa, Italy; E-Mails: laura.conte@unina2.it (L.C.); luigi.zeni@unina2.it (L.Z.); 2 Department of Physical Sciences, University of Naples “Federico II”, via Cinthia, Naples 80126, Italy; E-Mail: dmassarotti@na.infn.it; 3 Grup Optoelectronica si Componente Optice Integrate, Universitatea Tehnica, Cluj-Napoca 400114, Romania; E-Mail: Ramona.Galatus@bel.utcluj.ro; 4 CNR-IREA, via Diocleziano 328, 80124 Naples, Italy

**Keywords:** plastic optical fiber, Surface Plasmon Resonance

## Abstract

In silica optical fiber Surface Plasmon Resonance (SPR)-based sensors, an increase in fiber core diameter produces a corresponding increase in the sensitivity and Signal to Noise Ratio (SNR). In Plastic Optical Fiber (POF) realized in PMMA there are different influences of design parameters on the performance, as both sensitivity and SNR are concerned. In particular, the SNR, for different refractive index values of the analyte, in a 250 μm diameter POF is greater than the one in 1,000 μm diameter POF. On the other hand, the sensitivity, for the same refractive index values of the analyte, in a 1,000 μm diameter POF is greater than the one in a 250 μm diameter POF. The results of an experimental analysis demonstrating the above behavior are reported.

## 1. Introduction

Low cost sensors based on Surface Plasmon Resonance (SPR) in a Plastic Optical Fiber (POF) for biosensor implementation constitute a very sensitive technique for determining small refractive index changes at the interface between a metallic layer and a dielectric medium (analyte). This technique is widely used as a detection principle for many sensors used in the areas of biological and chemical sensing. Biological targets are generally transported through a microfluidic system by a buffer fluid or a carrier fluid. With SPR sensors, when the transducing media (ligands) react with the target molecules present in the analyte, the refractive index at the surface changes, and this change is detected by optical interrogation [1,2]. In many experimental setup, the SPR sensor system is based on a high refractive index prism coated with a thin metallic layer. The incidence angle of the light is varied in a wide range and surface plasma waves (plasmons) may exist whatever the surrounding medium, *i.e.*, a gas or a liquid. Nevertheless, the sensors are usually bulky, require expensive optical equipment, they aren't miniaturized and remote sensing seems to be quite difficult to implement. Furthermore, the proposed sensor, because of the small size and non-invasive features, can be used for medical (self-) diagnostics with the possibility of integration of SPR sensor platforms with optoelectronic devices eventually leading to “lab on a chip” and the use of an optical fiber allows remote sensing and may reduce the cost and dimensions of the device.

Due to the propagation of the light in the fiber, the angle of incidence on the metallic layer exceeds the critical angle which depends on the refractive indexes of both core and cladding [3]. Therefore SPR only exists for surrounding dielectrics whose refractive index lies in a narrow range. To overcome this drawback, Jorgenson *et al.* [2] used a polychromatic light source and a spectrograph. This device is low cost, easy to implement and can offer some attractive advantages such as the possibility of its use in presence of flammable substances and human hazardous environments because of its electricity-free, remote sensing capabilities.

In the above scenario, sensors based on bent or straight Plastic Optical Fiber (POF) represent a simple approach to low cost bio-sensing [4–7]. Recently, we have developed a new geometry for low cost sensor systems based on SPR in POF [5,7] with two attractive features, enabling it to be a candidate for successful biosensors implementation: it works with a planar gold layer and an external medium refractive index ranging from 1.332 to 1.418. The device has been successfully employed for celiac disease antibody detection [8]. In this paper we have analyzed the influence of POF core diameter on sensor performances.

## 2. Definitions and Samples Preparation

### 2.1. SPR Phenomenon

In the optical phenomenon of Surface Plasmon Resonance, a metal-dielectric interface supports a p-polarized electromagnetic wave, namely the Surface Plasmon Wave (SPW), which propagates along the interface. When the p-polarized light is incident on this metal-dielectric interface in such a way that the propagation constant (and energy) of resultant evanescent wave is equal to that of the SPW, a strong absorption of light takes place as a result of transfer of energy and the output signal demonstrates a sharp dip at a particular wavelength known as resonance wavelength. The so-called resonance condition is given by following expression [9,10]:

(1) $$\begin{array}{cc} K_{0}n_{c}\sin\vartheta =K_{0}{\left(\frac{{ɛ}_{mr}n_{s}^{2}}{{ɛ}_{mr}+n_{s}^{2}}\right)}^{1/2}; & K_{0}=\frac{2\pi }{\lambda } \end{array}$$

The term on the left-hand side is the propagation constant (K_inc_) of the evanescent wave generated as a result of Attenuated Total Reflection (ATR) of the light incident at an angle θ through a light coupling device (such as prism or optical fiber) of refractive index n_c_. The right-hand term is the SPW propagation constant (K_SP_); with ε_mr_ as the real part of the metal dielectric constant (ε_m_) and n_s_ as the refractive index of the sensing (dielectric) layer. This matching condition of propagation constants is heavily sensitive to even a slight change in the outer ambience; which makes this technique a powerful tool for sensing of different parameters.

In the following, for the characterization of sensors, we refer to a set of parameters (Sensitivity, Signal-to-Noise Ratio and Resolution) commonly usedin literature in the case of devices based on SPR in optical fibers, to allow an easy comparison of the respective performances [9,10].

In SPR sensors with spectral interrogation, the resonance wavelength (λ_res_) is determined with reference to the refractive index of the sensing layer (n_s_). If the refractive index of the sensing layer is altered by δn_s_, the resonance wavelength shifts by δλ_res_. The sensitivity (S_n_) of an SPR sensor with spectral interrogation is defined as [9,10]:

(2) $$S_{n}=\frac{\delta \lambda_{\text{res}}}{\delta n_{s}}\left[\frac{nm}{\text{RIU}}\right]$$

In other words, the sensitivity (S_n_) can be defined by calculating the shift in resonance wavelength per unit change in refractive index (nm/RIU). Owing to the fact that the vast majority of the field of an SPW is concentrated in the dielectric, the propagation constant of the SPW is extremely sensitive to changes in the refractive index of the dielectric. This property of SPW is the underlying physical principle of affinity SPR biosensors (Molecular Recognition Elements on the surface of metal recognize and capture analyte present in a liquid sample producing a local increase in the refractive index at the metal surface). The refractive index increase gives rise to an increase in the propagation constant of SPW propagating along the metal surface which can be accurately measured by optical means. The magnitude of the change in the propagation constant of an SPW depends on the refractive index change and its distribution with respect to the profile of the SPW field. If the binding occurs within the whole depth of the SPW field, the binding-induced refractive index change, produces a change in the real part of the propagation constant, which is directly proportional to the refractive index change. The binding-induced change in the propagation constant of the SPW is proportional to the refractive index change and the depth of the area within which the change occur.

The Signal-to-Noise Ratio of an SPR sensor depends on how accurately and precisely the sensor can detect the resonance wavelength and hence, the refractive index of the sensing layer. This accuracy in detecting the resonance wavelength further depends on the width of the SPR curve.

The narrower the SPR curve, the higher the detection accuracy. Therefore, if δλ_SW_ is the spectral width of the SPR response curve corresponding to some reference level of transmitted power, the detection accuracy of the sensor can be assumed to be inversely proportional to δλ_SW_. The signal-to-noise ratio of the SPR sensor with spectral interrogation is, thus, defined as [9,10]:

(3) $$\text{SNR}\left(n\right)={\left[\frac{\delta \lambda_{\text{res}}}{\delta \lambda_{\text{SW}}}\right]}_{n}$$

where δλ_SW_ can be calculated as the full width at half maximum of the SPR curve (FWHM). SNR is a dimensionless parameter strongly dependent on the refractive index changes. The resolution (Δn) of the SPR-based optical sensor can be defined as the minimum amount of change in refractive index detectable by the sensor. This parameter definitely depends on the spectral resolution (δλ_DR_) of the spectrometer used to measure the resonance wavelength in a sensor scheme. Therefore, if there is a shift of δλ_res_ in resonance wavelength corresponding to a refractive index change of δn_s_, then resolution can be defined as [9,10]:

(4) $$\Delta n=\frac{\delta n_{s}}{\delta \lambda_{\text{res}}}\delta \lambda_{DR}$$

### 2.2. Optical Sensor Systems

The fabricated optical sensor system was realized removing the cladding of a plastic optical fiber along half circumference, spin coating on the exposed core a buffer of Microposit S1813 photoresist, and finally sputtering a thin gold film by using a sputtering machine.

We have realized two SPR sensors based on POFs (Model no. PMMA POF D.980/1000, manufactured by Luceat Spa, Torbole Casaglia (BS), Italy). In the first case, the plastic optical fiber has a PMMA core of 980 μm and a fluorinated polymer cladding of 20 μm. In the second case, the plastic optical fiber has a PMMA core of 240 μm and a fluorinated polymer cladding of 10 μm.

**Figure 1.**
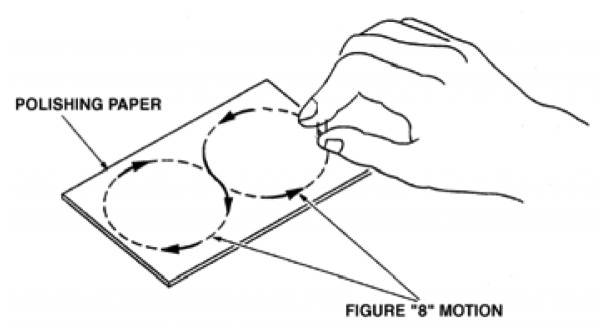
Figure “8” pattern for POF polishing.

The sample consisted in a plastic optical fiber (in the first case 250 μm diameter and in the second case 1000 μm) without jacket embedded in a resin block, with the purpose of easing the polishing process. The polishing process was carried out with a 5 μm polishing paper in order to remove the cladding and part of the core. For the POF with a 1,000 μm diameter, after 20 complete strokes with a figure “8” pattern (according to the manufacturer recommendations, as shown in Figure 1) in order to completely expose the core, a 1 μm polishing paper was used for another 20 complete strokes with a figure “8” pattern. The realized sensing region was about 10 mm in length. For the POF with a 250 μm diameter, after 10 complete strokes with a “Figure 8” pattern in order to completely expose the core, a 1 μm polishing paper was used for another 10 complete strokes with a figure “8” pattern. The realized sensing region was about 10 mm in length. It is important to underline that the above described manual polishing procedure causes unavoidable small differences in the final fiber core diameter, when different samples are prepared. Anyway, it has been experimentally verified that, due to the large core diameter, the sensors performances are not affected by the sample preparation procedure.

The buffer of Microposit S1813 photoresist was realized by using a spin coater for both POF (with 250 μm and 1,000 μm outer diameter, respectively). The Microposit S1813 photoresist is deposited in one drop (about 0.1 mL) on the center of the substrate. The sample is then spun at 6,000 rpm for 60 seconds. The final thickness of photoresist buffer was about 1.5 μm.

Finally, a thin gold film was sputtered by using a sputtering machine (Bal-Tec SCD 500). The sputtering process was repeated three times with a current of 60 mA for 35 seconds (20 nm for step). The gold film so obtained was 60 nm thick and presented a good adhesion to the substrate, verified by its resistance to rinsing in de-ionized water. The refractive indexes of the materials, in the visible range of interest, are about 1.49 for PMMA, 1.41 for fluorinated polymer and 1.61 for Microposit S1813 photoresist.

## 3. Experimental Setup

**Figure 2.**
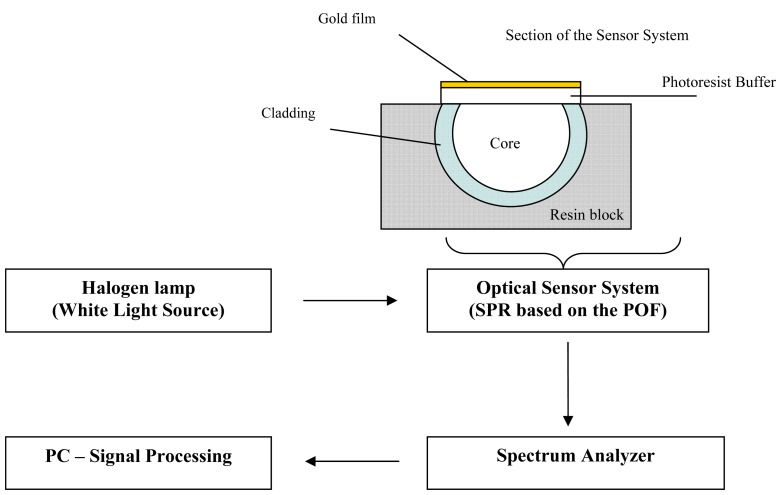
Experimental setup for SPR based on POF.

The experimental setup was arranged to measure the transmitted light spectrum and was characterized by a halogen lamp, illuminating the optical sensor systems (in the first case with a POF of 250 μm in diameter and in the second case a POF of 1,000 μm), and a spectrum analyzer, as shown in Figure 2. The employed halogen lamp (Model no. HL-2000-LL, manufactured by Ocean Optics) exhibits a wavelength emission range from 360 nm to 1,700 nm, while the spectrum analyzer detection range was from 200 nm to 850 nm. An Ocean Optics “USB2000+UV-VIS” spectrometer has been employed. The spectral resolution (δλ_DR_) of the spectrometer was 1.5 nm (FWHM). The spectrometer was finally connected to a computer.

The SPR curves along with data values were displayed online on the computer screen and saved with the help of advanced software provided by Ocean Optics.

## 4. Experimental Results

**Figure 3.**
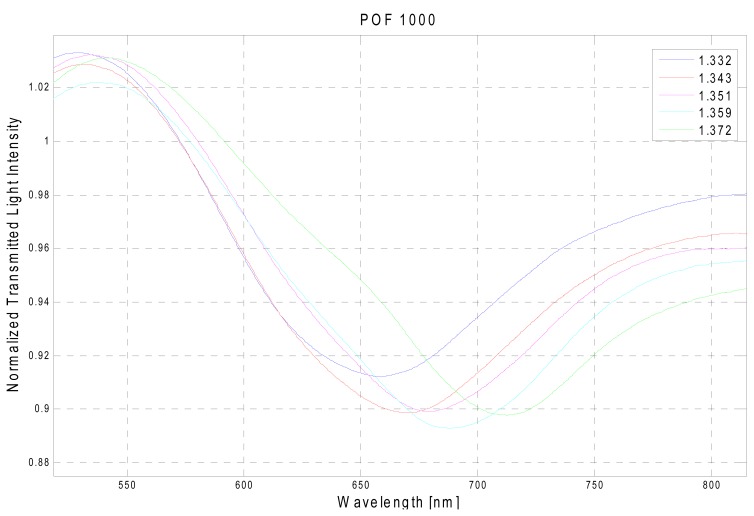
Experimentally obtained SPR transmission spectra, normalized to the air spectrum, for different refractive index of the aqueous medium. Configuration with a 1,000 μm diameter POF.

The presented experimental results are obtained by measuring SPR transmission spectra, normalized to the spectrum achieved with air as the surrounding medium, for different refractive indexes of the aqueous medium. The observed absorption band is the result of the convolution of different resonance peaks [9]. Each peak is obtained for a specific resonance condition defined by a given angle-wavelength couple [9,10] and water-glycerin solutions were used to achieve an aqueous medium with variable refractive index. Figure 3 reports the experimentally obtained SPR transmission spectra, in the case of 1,000 μm diameter POF, normalized to the spectrum achieved with air as the surrounding medium [11], for five different water-glycerin solutions with refractive index ranging from 1.332 to 1.372.

**Figure 4.**
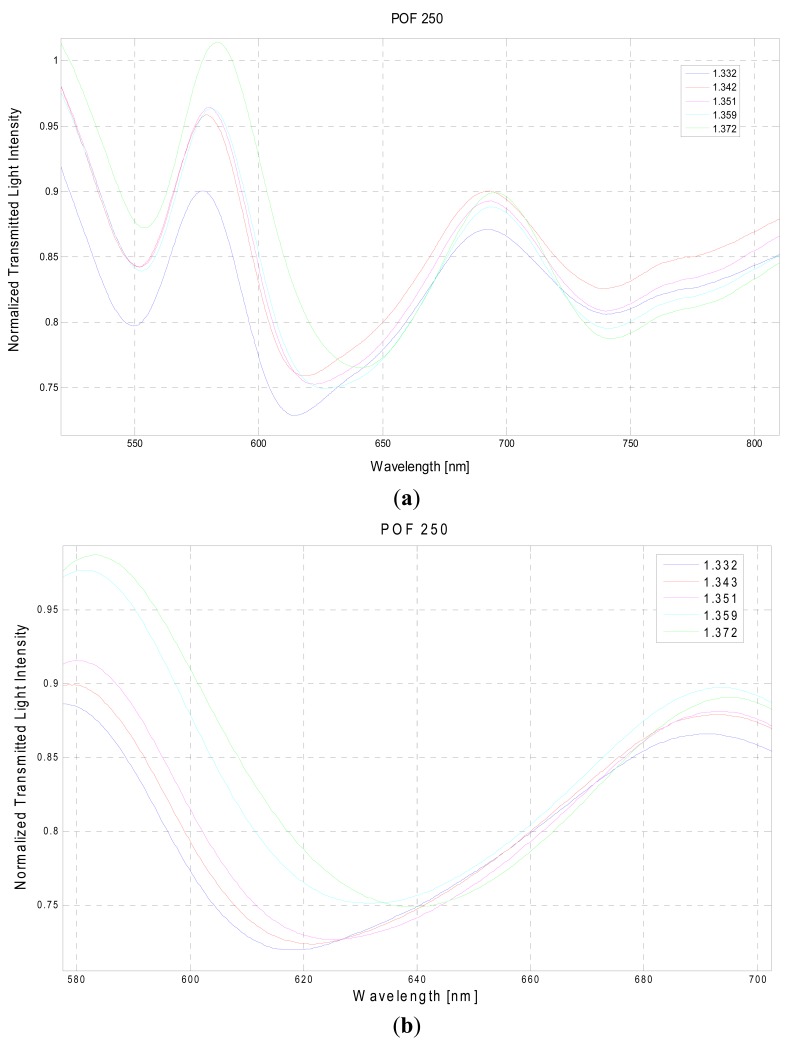
Configuration with a 250 μm diameter POF. (**a**) Experimentally obtained SPR transmission spectra, normalized to the air spectrum, for different refractive indexes of the aqueous medium. (**b**) Detail of the wavelengths range of interest.

In Figure 4 are presented the experimentally obtained SPR transmission spectra, normalized to the spectrum achieved with air as the surrounding medium, obtained with the 250 μm diameter POF.

**Figure 5.**
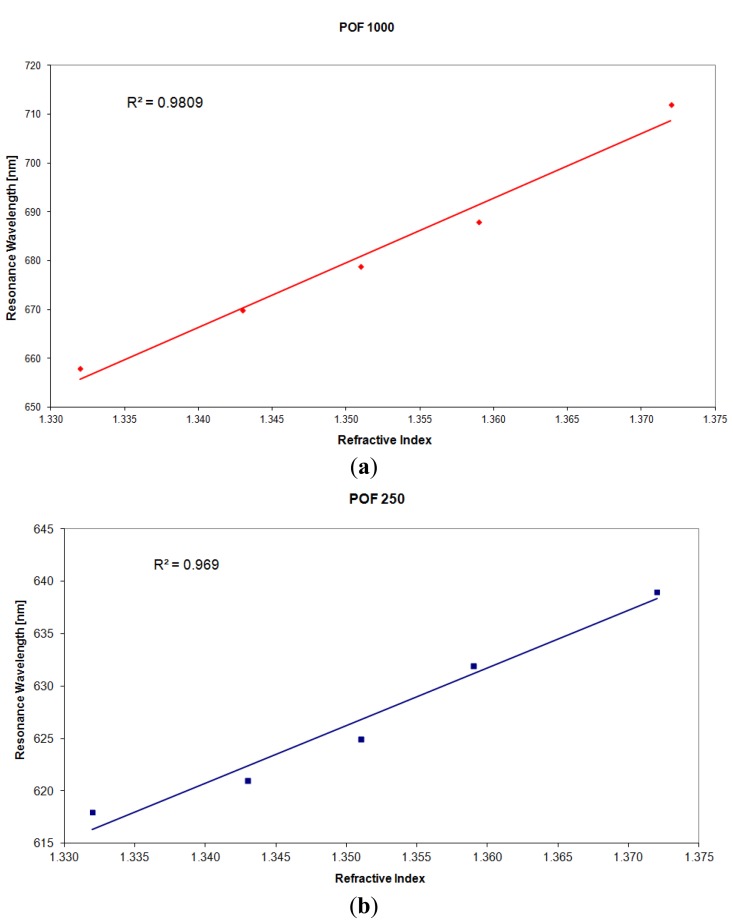
Plasmon resonance wavelength as a function of the refractive index. (**a**) Configuration with a 1,000 μm diameter POF. (**b**) Configuration with a 250 μm diameter POF.

Figure 5 shows the resonance wavelength *versus* the refractive index obtained with the two different configurations.

In the same figure is also presented the linear fitting to the experimental data, showing a good linearity for the sensors. The Pearson's correlation coefficient (R) is 0.99 for the sensor with a POF of 1,000 μm and 0.98 for the sensor with a POF of 250 μm diameter.

The sensitivity is the shift of the resonance wavelength (nm) per unit change in refractive index (nm/RIU). Therefore, it is the angular coefficient of the linear fitting. Figure 5 shows as the sensitivity increases with the fiber core diameter.

## 5. Discussion

Before entering the details of the discussion, as a similar analysis is present in the literature with reference to a sensor based SPR in silica optical fiber without any buffer layer between the fiber core and the gold film [10], it is convenient to briefly recall some fundamental aspects of light rays propagation in optical fibers where surface plasmons are excited.

Inside an optical fiber, any light ray making an angle θ from the normal to core-cladding interface undergoes multiple reflections (N_ref_), depending on the length of SPR sensing region (L) and fiber core diameter (D), according to the following relation [10,12]:

(5) $$N_{\text{ref}}(\theta )=\frac{L}{D\tan\theta }$$

To determine the effective transmitted power, the reflectance (R_e_) for a single reflection is raised to the power equal to corresponding number of reflections. Therefore, the generalized expression (all guided rays) for the normalized transmitted power (P_trans_) in sensors based on SPR in fiber optic can be written as:

(6) $$P_{\text{trans}}=\frac{\int_{\theta cr}^{\pi /2}{R_{e}}^{N_{\text{ref}}(\theta )}I(\theta )d\theta }{\int_{\theta cr}^{\pi /2}I(\theta )d\theta }$$

In Equation (6), I(θ) is the angular intensity distribution corresponding to the light source used. Further, θ_cr_ is the critical angle of the fiber, which heavily depends on the Numerical Aperture (NA) of the fiber and light wavelength.

**Figure 6.**
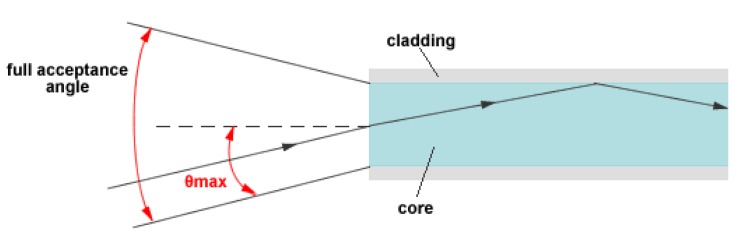
Optical fiber acceptance cone.

The angular range from θ_cr_ to π/2 covers whole range of guided rays (or modes) as these angles correspond to the highest order mode and the fundamental mode of an optical fiber, respectively. The number of modes that can propagate in a fiber depends on the fiber's Numerical Aperture (or acceptance angle) as well as on its core diameter and the wavelength of the light. For a step-index multimode fiber, the number of such modes, M, is approximated (M ≫ 1) by:

(7) $$M≅0.5*{\left(\frac{\pi *D*NA}{\lambda }\right)}^{2}$$

where D is the core diameter, λ is the operating wavelength, NA is the Numerical Aperture (or acceptance angle), see Figure 6.

In general, numerical aperture of a Plastic Optical Fiber is greater than that of a Silica Optical Fiber. The resonance condition (see Equation (1)) is satisfied at different wavelengths depending on which combination of core diameter and sensing region length is considered. It is so clear that the performance parameters of a fiber optic SPR sensor strictly depend on the values of design parameters such as fiber core diameter (D), sensing region length (L), and numerical aperture (NA). In the present work, we analyze the influence of two values of Plastic Optical Fiber core diameter (D) on the performance of a sensor based on Surface Plasmon Resonance in a POF, where the sensing region length is fixed and a photoresist buffer layer is placed between the fiber core and the gold film.

**Figure 7.**
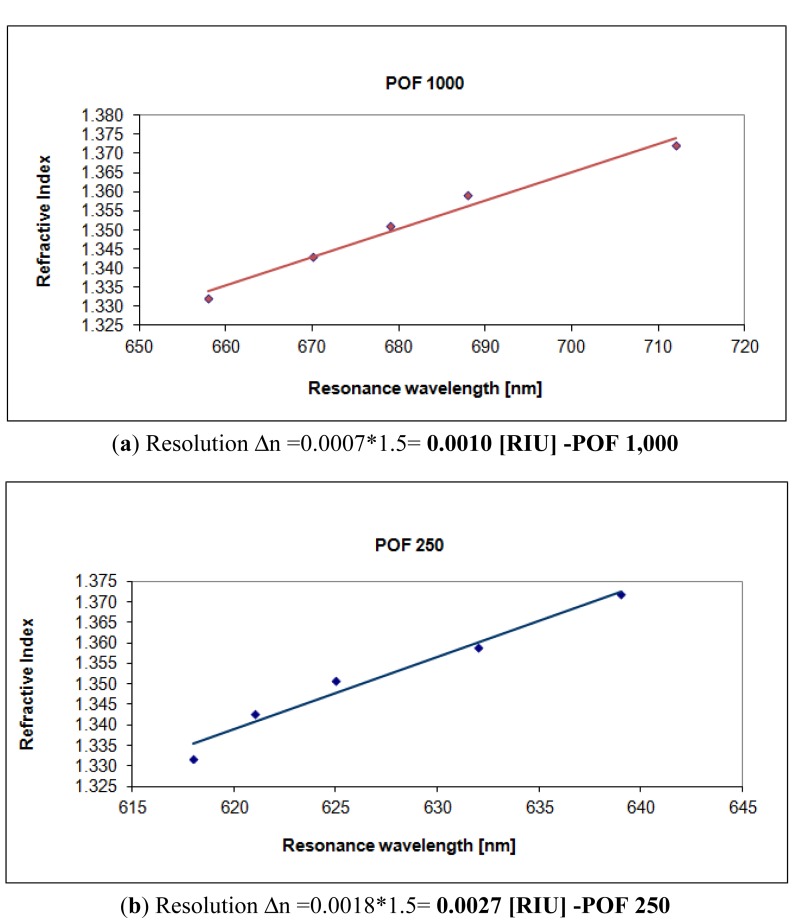
Refractive index as a function of the plasmon resonance wavelength. (**a**) Configurations with a 1,000 μm diameter POF. (**b**) Configurations with a 250 μm diameter POF.

For sensors based on SPR in optical fiber (silica or plastic) the shift in resonance wavelength (δλ_res_), for a fixed refractive index variation (δ_ns_), increases with a decrease in the number of reflections. Therefore, sensitivity increases with the increase of fiber core diameter and with the decrease of sensing region length. Furthermore, as sensor's resolution also depends on the variation of δλ_res_, therefore, similarly to sensitivity, resolution also tends to improve for larger fiber core diameters (see Figure 7). In fact, resolution (Δn) can be calculated as the angular coefficient of the linear fitting in Figure 7 multiplied to the spectral resolution (δλ_DR_) of the spectrometer used to measure the resonance wavelength.

**Figure 8.**
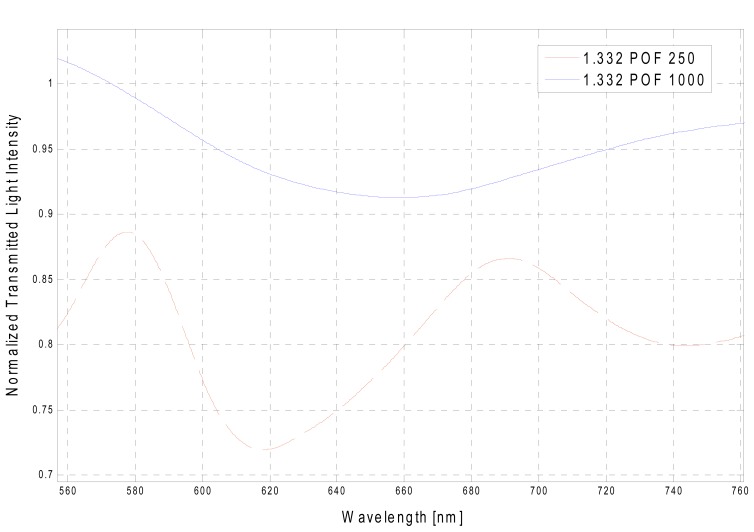
The full width at half maximum of the SPR curve for the two sensors configurations (250 μm and 1,000 μm POF diameter) for an external refractive index of 1.332.

The experimental results obtained with the two values of POF core diameter have shown as the Numerical Aperture of POF and the photoresist buffer layer have produced a different behavior with respect to many different configurations based on SPR in silica optical fibers, as SNR is concerned. From our experimental results, it is clear that the shift in resonance wavelength (δλ_res_), for a fixed refractive index variation (δ_ns_), increases when the core diameter increases. Therefore, sensitivity increases with an increase in fiber core diameter. Furthermore, in the sensors based on SPR in POF (configuration with the photoresist buffer layer) as already established, SPR curve width (δλ_SW_) increases with an increase in fiber core diameter. Therefore, it can be conveniently established that SPR curve width increases (δλ_SW_) with the increase of fiber core diameter, as shown in Figure 8 for a refractive index equal to 1.332.

**Figure 9.**
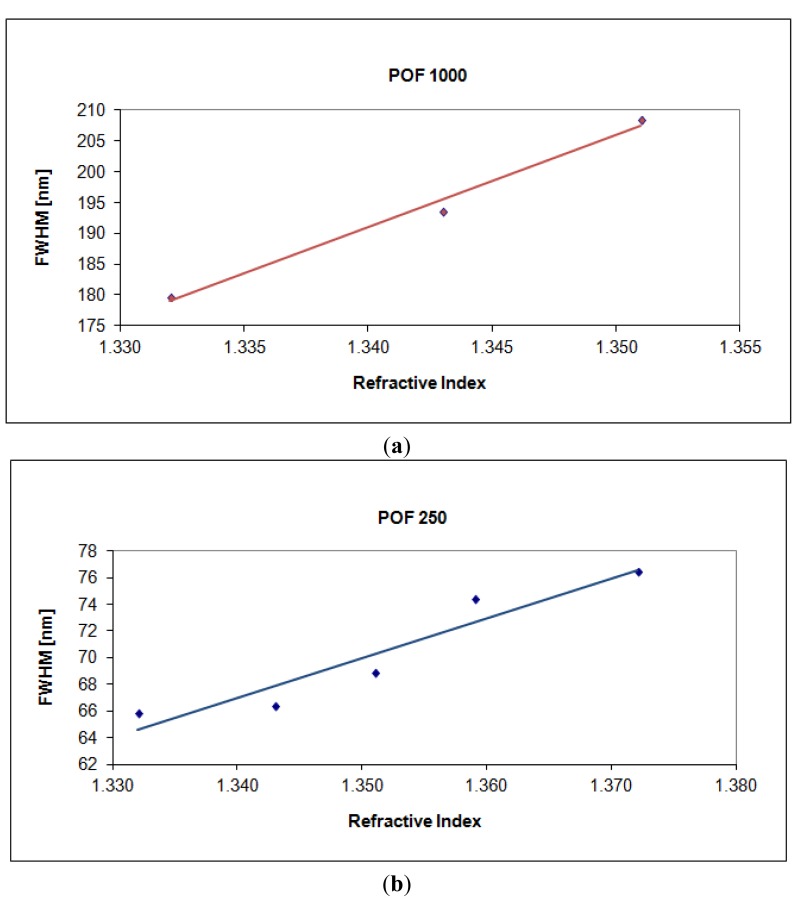
The full width at half maximum of the SPR curve as a function of the refractive index. (**a**) Configuration with a 1,000 μm diameter POF. (**b**) Configuration with a 250 μm diameter POF.

SPR curve width δλ_SW_ can be calculated as the full width at half maximum (FWHM) of the SPR curve. FWHM of the SPR curve as a function of the refractive index is shown in Figure 9. Therefore, SNR is expected to decrease because an increase in the shift in resonance wavelength (δλ_res_) produces a larger increase in curve width (δλ_SW_), for a fixed increase in fiber core diameter. This result is also evident in Figures 3 and 4.

More precisely, for a POF with 250 μm of diameter the angular coefficient of the linear fitting shown in Figure 5 (δλ_res_) is greater than the angular coefficient of the linear fitting shown in Figure 9 (δλ_SW_). In this case SNR is greater than one. For a POF with 1,000 μm of diameter the angular coefficient of the linear fitting shown in Figure 5 (δλ_res_) is lower than the angular coefficient of the linear fitting shown in Figure 9 (δλ_SW_). In this case SNR is less than one.

**Figure 10.**
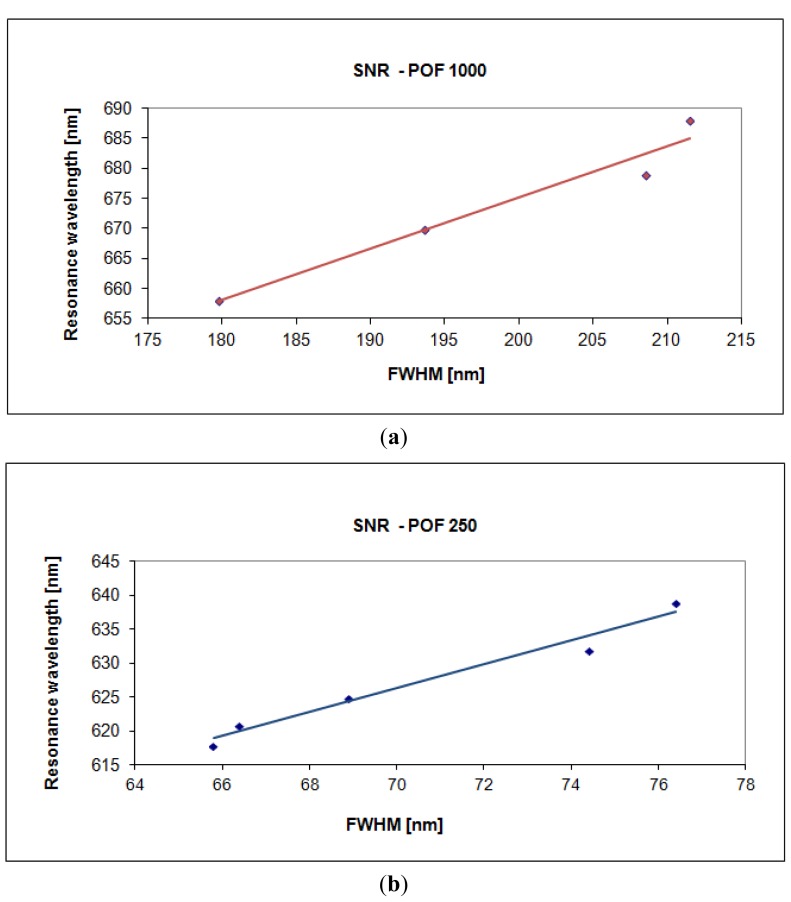
Plasmon resonance wavelength as a function of the full width at half maximum of the SPR curve. (**a**) Configuration with a 1,000 μm diameter POF. (**b**) Configuration with a 250 μm diameter POF.

The plasmon resonance wavelength as a function of the full width at half maximum of the SPR curve is shown in Figure 10. SNR can be calculated as the angular coefficient of the linear fitting reported in Figure 10. From the above figure, it is clear that the SNR increases when the fiber core diameter decreases. It is important to emphasize that the calculation, from experimental data, of the single values of above parameters has been carried out by employing a first-order approach, while the linear fitting does not imply an actual linear relationship but it is just a way to extrapolate a trend and allow an easy comparison between the two sensor systems [9,10,12,13].

For a clearer comparative analysis between the two sensors with 250 μm and 1,000 μm diameter POFs, Table 1 summarizes the averages values of the experimentally measured performance parameters, evaluated by Matlab software, for external medium refractive index ranging from 1.332 to 1.372.

## 6. Conclusions and Future Trends

Two sensors based on SPR in plastic optical fiber (with a buffer layer between fiber core and gold film), have been realized and experimentally tested. The proposed devices are based on the excitation of surface plasmons at the interface between under test medium and a thin gold layer deposited on a photoresist buffer spin coated on the plastic fiber core. The sensing devices have been characterized by exploiting a halogen lamp to illuminate the optical fiber and observing the transmitted spectra, normalized to the spectrum transmitted when the outer medium is air.

The experimental results indicate that the configuration with a fiber diameter of 1,000 μm exhibits better performance in terms of sensitivity and resolution but not in terms of SNR. On the contrary, in our case SNR increases with a decrease in fiber core diameter. Work is in progress to extend the analysis to a larger number of POF diameters.

## Figures and Tables

**Table 1.** Performance comparison for the two sensors configurations: 250 μm and 1,000 μm diameter POF, respectively.

**POF Diameter [μm]**	**Resolution (Δn) [RIU]**	**Signal-to-noise ratio (SNR)**	**Sensitivity (S_n_) [nm/RIU]**	**FWHM/Δn [nm/RIU]**
250	0.0027	1.7548	0.549 × 10^3^	0.298 × 10^3^
1,000	0.0010	0.8569	1.325 × 10^3^	1.495 × 10^3^

## References

[1] Homola J. (2003). Present and future of surface plasmon resonance biosensors. Anal. Bioanal. Chem..

[2] Jorgenson R.C., Yee S.S. (1993). A fiber-optic chemical sensor based on surface plasmon resonance. Sens. Actuators B: Chem..

[3] Trouillet A., Ronot-Trioli C., Veillas C., Gagnaire H. (1996). Chemical sensing by surface plasmon resonance in a multimode optical fibre. Pure Appl. Opt..

[4] Munoz-Berti V.M., López-Pérez A.C., Alén B., Costa-Krämerm J.L., García-Martín A., Lomer M., López-Higuera J.M. (2010). Low cost plastic optical fiber sensor based on surface plasmon resonance. Proc. SPIE.

[5] Cennamo N., Massarotti D., Conte L., Zeni L. SPR in Plastic Optical Fibers: A Simple Geometry for Low-Cost Biosensors.

[6] Bartlett R.J., Philip-Chandy R., Eldridge P., Merchand D.F., Morgan R., Scully P.J. (2000). Plastic optical fibre sensors and devices. Trans. Inst. Meas. Control.

[7] Cennamo N., Massarotti D., Conte L., Zeni L. (2011). Low cost sensors based on SPR in a plastic optical fiber for biosensor implementation. Sensors.

[8] Cennamo N., Varriale A., Pennacchio A., Staiano M., Massarotti D., Zeni L., D'Auria S. (2013). An innovative plastic optical fiber-based biosensor for new bio/applications. The Case of Celiac Disease. Sens. Actuators B: Chem..

[9] Kanso M., Cuenot S., Louarn G. (2008). Sensitivity of optical fiber sensor based on surface plasmon resonance: Modeling and experiments. Plasmonics.

[10] Dwivedi Y.S., Sharma A.K., Gupta B.D. (2008). Influence of design parameters on the performance of a SPR based fiber optic sensor. Plasmonics.

[11] Iga M., Sek A., Watanabe K. (2005). Gold thickness dependence of spr-based hetero-core structured optical fiber sensor. Sens. Actuators B: Chem..

[12] Anuj K., Sharma R.J., Gupta B.D. (2007). Fiber-optic sensors based on Surface Plasmon Resonance: A comprehensive review. IEEE Sens. J..

[13] Weber W.H., McCarthy S.L., Ford G.W. (1974). Perturbation theory applied to gain or loss in an optical waveguide. Appl. Opt..

